# Commentary: Miniaturized extracorporeal circulation, the “circuit-of-choice?”

**DOI:** 10.1016/j.xjon.2021.11.006

**Published:** 2021-11-06

**Authors:** Hong Rae Kim, Joon Bum Kim

**Affiliations:** Department of Thoracic and Cardiovascular Surgery, Asan Medical Center, University of Ulsan College of Medicine, Seoul, Republic of Korea

**Figure:**
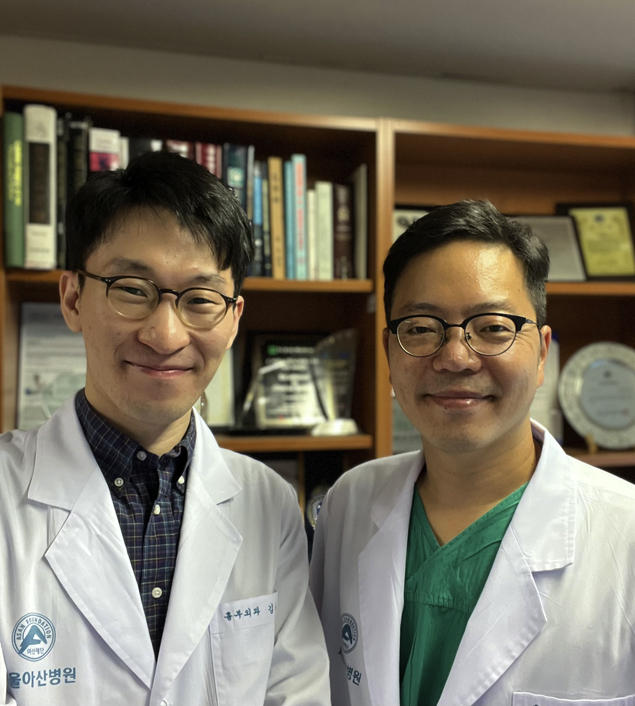
Hong Rae Kim, MD (*left*), and Joon Bum Kim, MD, PhD (*right*)

**Central Message**

The analysis demonstrates significant superiority of MECC in reducing the composite incidence of postoperative mortality, stroke, renal failure, and myocardial infarction over CECC.

See Article page 418.

Cardiopulmonary bypass (CPB), a prerequisite for major cardiac surgery, inevitably accompanies hemodilution and systemic inflammatory response syndrome that may contribute to impairments in the O_2_-carrying capacity, increased transfusion requirements, and multiorgan dysfunction, leading to increased risks of morbidity and mortality.^1^ In addition, cardiotomy suction and blood air contact in a venous reservoir can also cause hemolysis and coagulopathy.

Miniaturized extracorporeal circulation (MECC) has been developed to minimize the drawbacks of CPB by attenuating systemic inflammatory response syndrome via limiting the blood–air interface and reducing the artificial tubing length with the renunciation of cardiotomy suction and a venous reservoir.^2^ Most previous clinical studies, including several meta-analyses, have shown the potential benefits of MECC over conventional extracorporeal circulation (CECC)^3^^,^^4^; however, these studies have failed to demonstrate overt benefits in terms of clinically relevant hard end points and also were not supported by subsequent large-scale studies. The use of MECC has therefore been still limited in clinical practice, with only 4% to 10% of operations using MECC.^5^ Even more, the existence of MECC itself may still be unfamiliar to some cardiac surgeons.

In this issue of the *Journal*, Cheng and colleagues^6^ sought to compare the outcomes between MECC and CECC through 42 randomized controlled trial studies that involved 4340 patients undergoing coronary arterial bypass graft or heart valve surgeries. The authors found that MECC significantly reduces a composite incidence of postoperative mortality, stroke, renal failure, and myocardial infarction as compared with CECC. In addition, patients in the MECC group showed less postoperative atrial fibrillation, shorter hospital and intensive care unit stays, less blood loss, and lower levels of interleukin-6 and interleukin-8.

Despite its potential superiority over CECC, MECC has not received general acceptance in clinical practices because of several lingering concerns. First, the closed-circuit nature of MECC may jeopardizes the safety buffer in the event of massive bleeding. Second, there is concern about its limited capacity of removing air bubbles from the circulating blood. Third, the use of cell savers instead of CPB suction systems may also lead to hemodilution and the need for heparinization of the recruited blood may result in more hemorrhagic conditions. Fourth, as the authors stated, the high cost of the MECC circuit may hamper its application to get general acceptance. Lastly, we believe insufficient comparative outcome data—demonstrating superiority of MECC—might have prevented its widespread applications.

The issue of MECC is of growing interest, and the present study well handled this issue through a large-scale meta-analysis. In light of the findings of this current study, there seems to be no reason not to use MECC in isolated valve surgeries as well as in coronary arterial bypass graft, provided that the aforementioned concerns are adequately addressed further. One limitation in the paper by Cheng and colleagues noticed is that although the analysis demonstrates significant superiority of MECC in reducing the composite incidence of postoperative mortality, stroke, renal failure, and myocardial infarction over CECC, the data could not confirm the results on each of the primary end points due to the limited numbers of each event. Despite these limitations, this comparative collective data on MECC and CECC will broaden our perspective in the area of CPB and contribute to the accumulation of evidence in this field. A further collection of clinical experiences and data analysis from the larger, well-designed randomized trials are warranted for a deeper understanding of this issue.
